# Retinal Cone Photoreceptors of the Deer Mouse *Peromyscus maniculatus*: Development, Topography, Opsin Expression and Spectral Tuning

**DOI:** 10.1371/journal.pone.0080910

**Published:** 2013-11-15

**Authors:** Patrick Arbogast, Martin Glösmann, Leo Peichl

**Affiliations:** Max Planck Institute for Brain Research, Frankfurt am Main, Germany; National Eye Institute, United States of America

## Abstract

A quantitative analysis of photoreceptor properties was performed in the retina of the nocturnal deer mouse, *Peromyscus maniculatus*, using pigmented (wildtype) and albino animals. The aim was to establish whether the deer mouse is a more suitable model species than the house mouse for photoreceptor studies, and whether oculocutaneous albinism affects its photoreceptor properties. In retinal flatmounts, cone photoreceptors were identified by opsin immunostaining, and their numbers, spectral types, and distributions across the retina were determined. Rod photoreceptors were counted using differential interference contrast microscopy. Pigmented *P. maniculatus* have a rod-dominated retina with rod densities of about 450.000/mm^2^ and cone densities of 3000 - 6500/mm^2^. Two cone opsins, shortwave sensitive (S) and middle-to-longwave sensitive (M), are present and expressed in distinct cone types. Partial sequencing of the S opsin gene strongly supports UV sensitivity of the S cone visual pigment. The S cones constitute a 5-15% minority of the cones. Different from house mouse, S and M cone distributions do not have dorsoventral gradients, and coexpression of both opsins in single cones is exceptional (<2% of the cones). In albino *P. maniculatus*, rod densities are reduced by approximately 40% (270.000/mm^2^). Overall, cone density and the density of cones exclusively expressing S opsin are not significantly different from pigmented *P. maniculatus*. However, in albino retinas S opsin is coexpressed with M opsin in 60-90% of the cones and therefore the population of cones expressing only M opsin is significantly reduced to 5-25%. In conclusion, deer mouse cone properties largely conform to the general mammalian pattern, hence the deer mouse may be better suited than the house mouse for the study of certain basic cone properties, including the effects of albinism on cone opsin expression.

## Introduction

The New World deer mice (Genus *Permomyscus* with more than 50 species) belong to the rodent family Cricetidae, subfamily Neotominae. *Peromyscus maniculatus* is the most common deer mouse species in the continental United States and thus has ecological relevance. Since 1916, *P. maniculatus* has been used as a laboratory rodent, particularly in toxicological and epidemiological research, as well as in ecological, behavioral, and genetic studies [1]. *Peromyscus maniculatus* may be a useful additional model to the house mouse *Mus musculus* also for retinal and particularly for photoreceptor studies [2]. The house mouse with its plethora of genetically modified lines has gained unsurpassed importance for research, but with respect to retinal research it has a drawback. Its cone photoreceptors have an opsin expression pattern that deviates from the one found in most other mammals including humans (reviews [3]:[4],), leaving concerns as to how representative findings on mouse photoreceptors are for mammals in general. We have analyzed the cone properties in the deer mouse retina to see whether they are closer to what is considered the basic mammalian blueprint.

The basic mammalian cone arrangement consists of two spectral cone types containing a shortwave-sensitive (S) and a middle-to-longwave-sensitive (M) visual pigment, respectively (reviews [3,6]:). Depending on author, the latter pigment is referred to as M, L, or M/L; here we use the term M. These two cone types are the basis for the dichromatic form of color vision most commonly found in non-primate mammals (reviews [6]:[7],). Commonly, the M cones form a majority, and the S cones a roughly 10% minority, of the cones. However, there are a number of species-specific variations on this common theme. In most mammals, the S pigment is tuned to violet/blue, but in some it is tuned to UV. Some species show regional differences in the mix of S and M cones, usually increased or even dominant S cone proportions in ventral retina. Some species have lost the S cones completely and are M cone monochromats. Finally, some species coexpress the M and S opsin in many of their cones. These variations have been the topics of many reviews (e.g., [3], [4] [6], [8,13],).

Rodents are the largest and most diverse mammalian order, they have adapted to a large variety of habitats and lifestyles, and species differences in photoreceptor arrangements are correspondingly large, including all the above variations (overviews [3]: [4], [7], [9], [12],). The house mouse is a striking example: its S pigment is tuned to UV, the dorsal retina has a normal mix of M and S cones, whereas most cones in ventral retina dominantly express the S opsin and coexpress lower levels of M opsin [14], [15]. Hence a specific rationale of the present study was to look at the photoreceptor properties of deer mouse in comparison to other rodents. To date, the photoreceptor properties of Neotominae have not been studied extensively. The present assessment of *P. maniculatus* photoreceptor properties focuses on the topographic distributions of the spectral cone types and the spectral tuning of the S pigment, the question of opsin coexpression in mature cones, the postnatal maturation of cone opsin expression, and potential photoreceptor differences between pigmented and albino strains. The results add to the database that identifies basic properties common to all rodents and group- or species-specific specializations.

## Materials and Methods

### Ethics Statement

All procedures for animal husbandry, breeding and killing complied with the NIH Principles of Laboratory Animal Care and the corresponding German laws. Animal husbandry and breeding were approved by the responsible local authority, the Veterinäramt Frankfurt am Main, on the basis of the German Animal Protection Law (Tierschutzgesetz, TSchG) §11. The study did not involve animal experiments as defined in the TSchG and did not require an ethics committee approval. Animals were killed by decapitation under deep isofluran anesthesia, in strict accordance with §4 TSchG (§4 Abs. 3: killing of vertebrates for scientific use).

 Animals and tissue preparation Male and female *Peromyscus maniculatus bairdii* (c+/c+, wildtype melanin pigmentation) were purchased from the Peromyscus Genetic Stock Center (PGSC) at the University of South Carolina and bred at the animal facility of the Max Planck Institute for Brain Research in Frankfurt, Germany. Nineteen animals, including males and females, of ages from postnatal day (P) 7 to serveral months were deeply anesthetized by isoflurane and decapitated. Because opsin expression levels change slightly over the day, all animals were killed between 9 am and 11 am. Immediately post mortem, eyes were marked at the dorsal pole for orientation, enucleated, punctured at the corneal rim for better fixative penetration, and immersion-fixed in 4% paraformaldehyde in 0.1 M phosphate buffer (PB, pH 7.4) for 4 - 6 h at room temperature. After a wash in PB, the retina was isolated from the eyecup and either processed immediately, or cryoprotected by successive immersion in 10%, 20% and 30% (w/v) sucrose in PB and frozen at -20°C for later use. Eyes of albino *P. maniculatus* (c/c, devoid of melanin pigment, tyrosinase activity absent) were collected at the PGSC, dissected and fixed following the same protocol as for *P. maniculatus bairdii*.

 For frozen vertical sections of the retina (i.e., perpendicular to the retinal layers), the tissue was cryoprotected by successive immersion in 10%, 20% and 30% (w/v) sucrose in PB, transferred to tissue-freezing medium (Reichert-Jung, Bensheim, Germany), sectioned at a thickness of 12 - 14 µm with a cryostat, and collected on slides. For the assessment of general retinal morphology, pieces of retina were embedded in Epon, sectioned vertically at 1µm and stained with toluidine blue.

### Immunohistochemistry

Immunostaining was performed on frozen sections and whole retinas. Before staining of whole retinas, the retina was cryoprotected in 30% sucrose in PB and repeatedly shock-frozen and thawed to improve penetration of the antibodies.

Opsin immunohistochemistry followed previously described protocols [16], [17]. Briefly, the tissue was preincubated for 1 h in PBS with 0.5% Triton X-100 and 10% normal donkey serum (NDS). Incubation in the primary antibody/antiserum solution was overnight at room temperature. Rod opsin was detected with the mouse monoclonal antibody rho4D2 (dilution 1:500). The cone opsins were assessed by double immunofluorescence labeling, detecting the middle-to-longwave-sensitive (M) cone opsin with the rabbit antiserum JH 492 (dilution 1:2000) and the shortwave-sensitive (S) cone opsin with the goat antiserum sc-14363 (dilution 1:500). Rho4D2 was kindly provided by R. S. Molday [18], JH 492 was kindly provided by J. Nathans [19] and sc-14363 was purchased from Santa Cruz Biotechnology Inc., Heidelberg, Germany. All these antibodies have been used in several previous studies to reliably label the respective opsins in a range of mammals including rodents (see, e.g., [20]-[26]). Binding sites of the primary antibodies were detected by indirect immunofluorescence, after a 1 h incubation of the sections in the secondary antiserum. For the rod opsin labeling Cy5-conjugated donkey anti-mouse IgG was used, for the cone opsin double-labeling a mixture of Alexa 488-conjugated donkey anti-goat IgG and Cy5-conjugated donkey anti-rabbit IgG. Omission of the primary antibodies from the incubation solution resulted in no staining. Whole retinas were flattened onto slides with the photoreceptor side up. All tissue was coverslipped with an aqueous mounting medium (AquaPoly/Mount, Polysciences Inc., Warrington, PA, USA). 

### Imaging and Analysis

Tissue was analyzed with a Zeiss Axioplan 2 microscope equipped with epifluorescence. Micrographs were taken with a CCD camera and the Axiovision LE software (Carl Zeiss Vision, Germany). The images were adjusted for brightness and contrast using Adobe Photoshop CS4.

Total photoreceptor (cone plus rod) densities were assessed in retinal wholemounts by focusing on the photoreceptor inner segments with differential interference contrast (DIC) optics, using a x63 oil immersion objective. M and S cone densities were assessed from double-immunofluorescence micrographs taken with a x40 objective. For the density maps of adult retinae, about 30 sampling points were suitably spaced across the retina; for the cone density graphs of younger ages, sampling points were spaced along the dorso-ventral axis of the retina. Photoreceptor densities were not corrected for shrinkage, because shrinkage was negligible in the tissue mounted with the aqueous medium. Density maps were created using the DeltaGraph 5.4 software.

### S opsin sequencing

Genomic DNA of wildtype and albino *P. maniculatus* was provided by the PGSC and used to PCR-amplify the S opsin gene from exon 1 to exon 3 using primers 5’-GGTGGGGCCCTGGGATGGGCCTCAG -3’ and 5’- ATGAAGAGGAACCAGGTGTAGTAC -3’. Reactions were conducted in 50 µl volumes on a MJ Mini Thermal Cycler (Bio-Rad, Hercules, CA, USA) with initial denaturation at 94°C for 3 min, denaturation at 94°C for 30 s, annealing at 64°C for 45 s, extension at 72°C for 45 s for 35 cycles, followed by a final extension at 72°C for 10 min. A single ca. 950 bp product was amplified, purified, and directly sequenced on both strands.

## Results

### Adult photoreceptor arrangements in the wildtype and albino deer mouse

**Figure 1:**
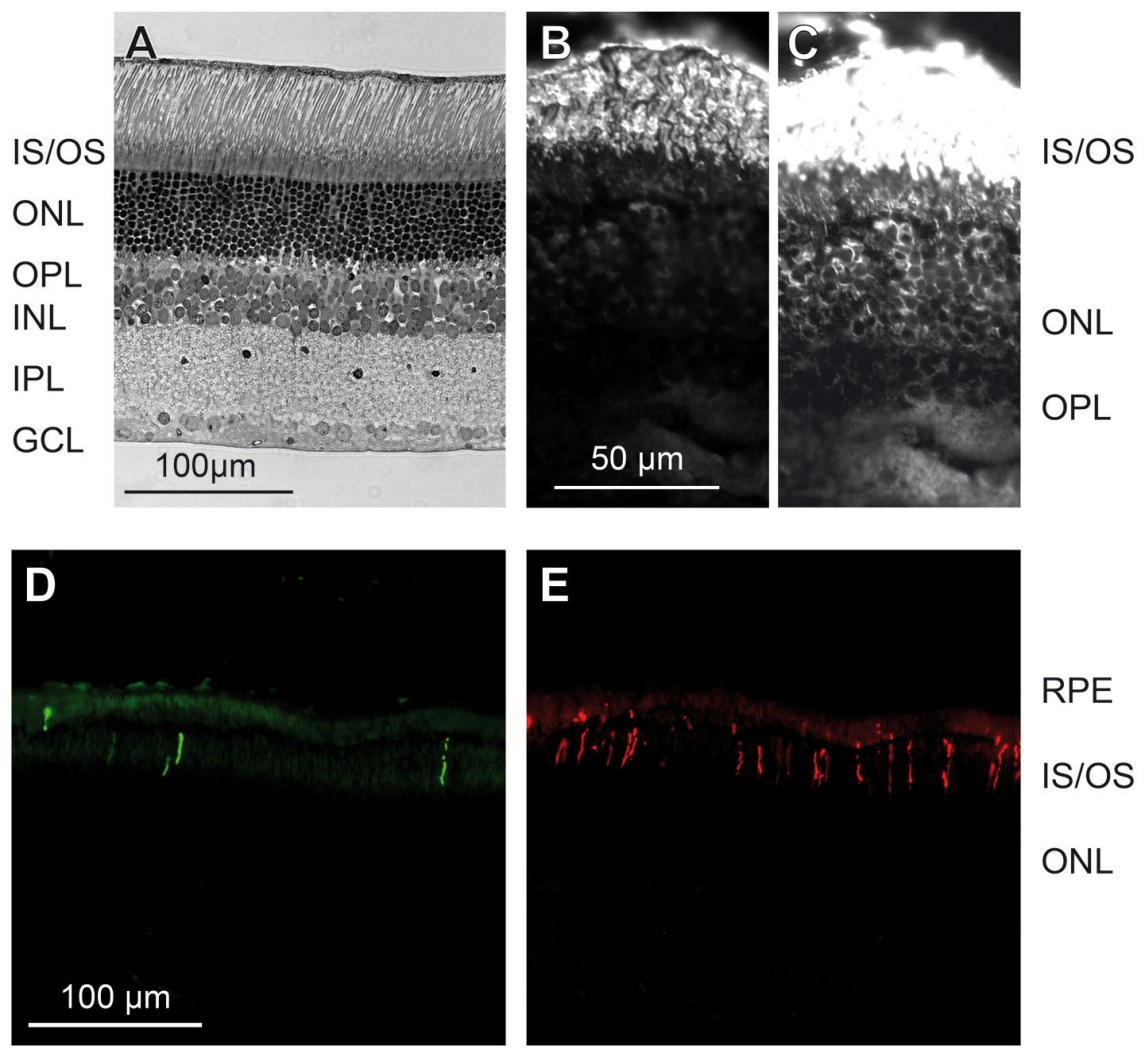
Deer mouse retinal morphology and photoreceptors. (**A**) Transverse 1µm section stained with toludine blue to show the retinal layers in *Peromyscus maniculatus*. (**B**) Vertical cryostat section immunolabeled for rod opsin, revealing a dense population of rod outer segments. (**C**) Same field as B, overexposed to show the weaker immunoreactivity of the rod somata. (**D**, **E**) Vertical cryostat section double-immunolabeled for S cone opsin (D) and M cone opsin (E), showing the outer segments of a substantial M cone population and a sparser S cone population. OS, IS, photoreceptor outer and inner segments; ONL, outer nuclear layer; OPL, outer plexiform layer; INL, inner nuclear layer; IPL, inner plexiform layer; GCL, ganglion cell layer; RPE, retinal pigment epithelium.

Transverse sections of the wildtype, pigmented *Peromyscus maniculatus* retina showed the typical mammalian layering with a retinal thickness of about 230 µm in central regions (Figure 1A). The outer nuclear layer (ONL), containing the photoreceptor somata, was the thickest layer with approximately 10 tiers of somata. This confirms the observations of Shupe et al. [2] and indicates a strongly rod-dominated retina. A high rod density was confirmed by immunolabeling for the rod opsin (Figure 1B, C). Immunolabeling also revealed the presence of both middle-to-longwave-sensitive (M) and shortwave-sensitive (S) cone opsins, with many more cones expressing the M than the S opsin (Figures 1D, E).

**Figure 2:**
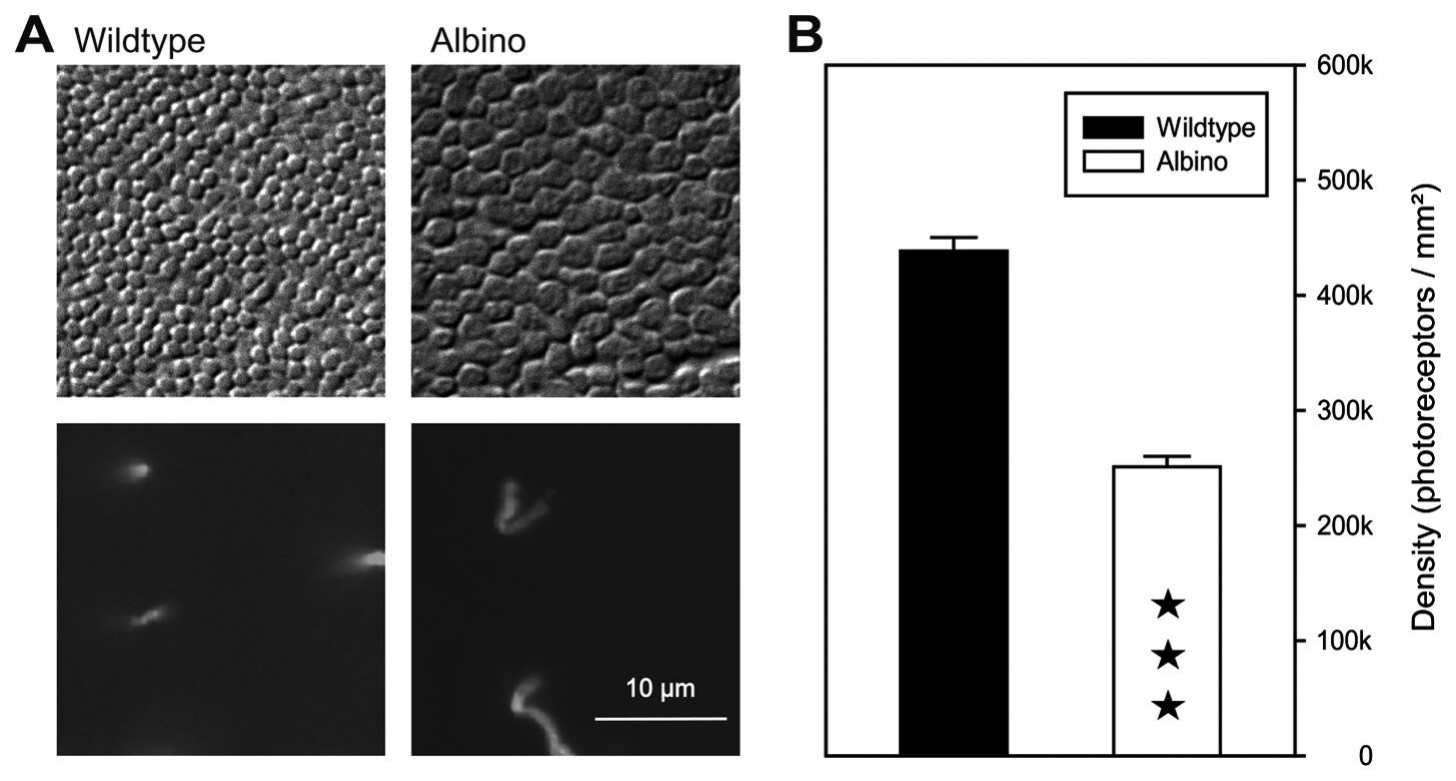
Photoreceptor differences between pigmented and albino deer mice. (**A**) *Top*: flat view of photoreceptor inner segments in wildtype and albino animals, showing the larger IS diameter and lower density of the albino photoreceptors, differential interference contrast images. *Bottom*: cones in the same fields, combined immunofluorescence labeling for M and S opsin. The scale bar applies to all images. (**B**) Quantification of the photoreceptor densities in wildtype and albino. Counts were made at several positions across the retina (wildtype: 11 positions in 1 retina; albino: 6 positions in 2 retinae of 2 individuals; data given as mean and SEM. ★★★, difference statistically significant at p<0,001 (t-test).

Retinal flatmounts were used to quantify photoreceptor densities. Total photoreceptor densities were assessed by differential interference contrast (DIC) microscopy in favorable patches of retina, where the photoreceptor inner and outer segments were oriented vertically (Figure 2A). In wildtype deer mice, photoreceptor densities were in the range of 400,000-520,000/mm^2^ (Figure 2B), showing no consistent regional variation across the retina. In albino deer mice, the individual photoreceptors where noticeably larger in diameter (Figure 2A) resulting in a 40% lower density of 220,000-320,000/mm^2^ (Figure 2B). Given the low cone densities of *P. maniculatus* (see Figure 2A and below), the above values can also be taken as the rod density range.

**Figure 3:**
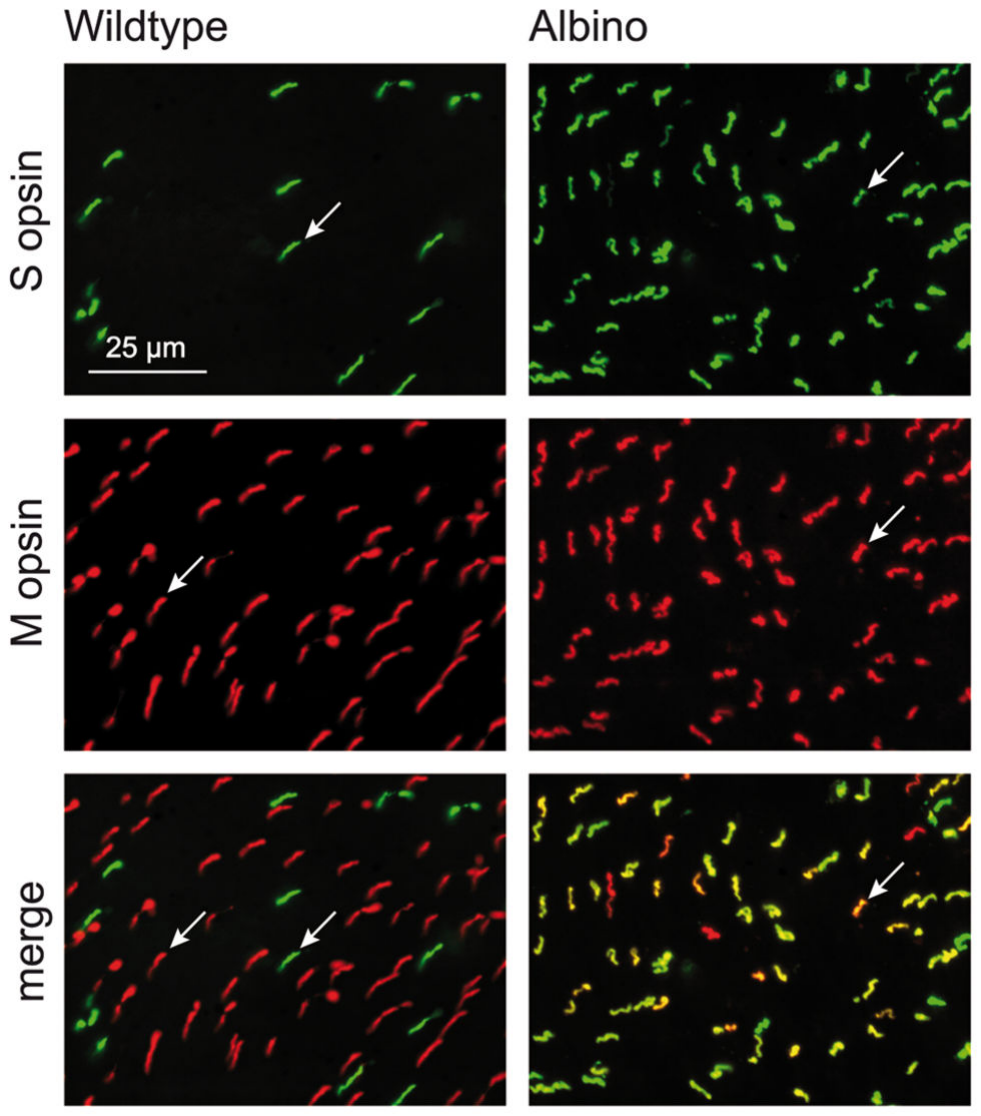
Cone opsin expression in adult pigmented and albino deer mice. Double immunofluorescence labeling for S and M opsin in flatmounted retinae, the focus is on the opsin-containing cone outer segments. **Top**: There is a sparse population of S opsin-expressing cones in the wildtype and a more numerous one in the albino. **Middle**: The populations of M opsin-expressing cones are similar in both genotypes. **Bottom**: Merge of the top and middle images shows that S and M cones form separate populations in the wildtype, whereas many cones coexpress both opsins in the albino (yellowish colors); cone examples are arrowed. The scale bar applies to all images.

**Figure 4:**
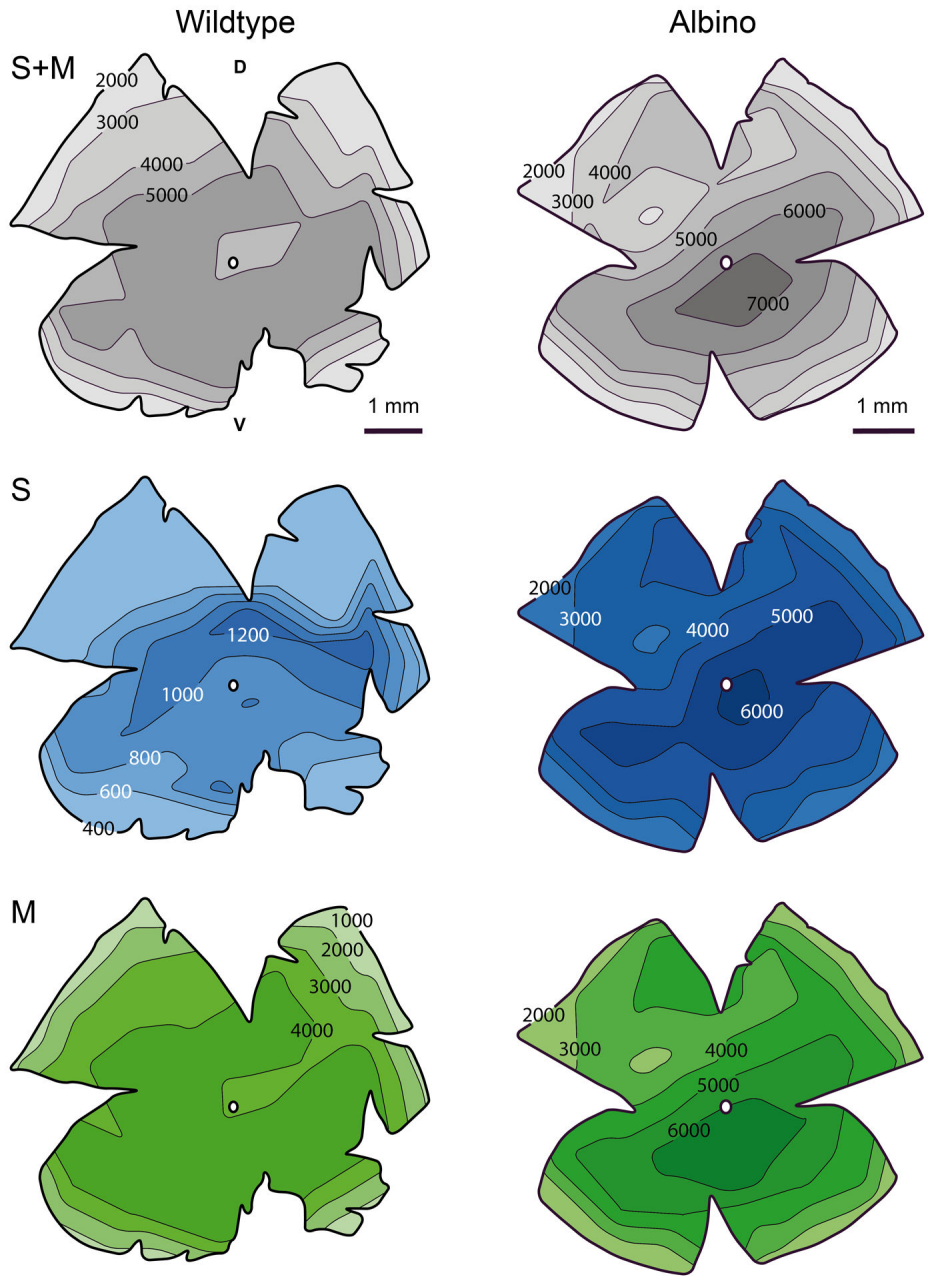
Maps of cone densities in adult pigmented and albino deer mice. The two columns each show three maps of the same retina, giving total cone density (*top*), S cone density (*middle*), and M cone density (*bottom*). Densities at the isodensity lines are cones/mm². The small circles in the center of the retinae indicate the optic nerve head. D, dorsal; V, ventral.

**Figure 5:**
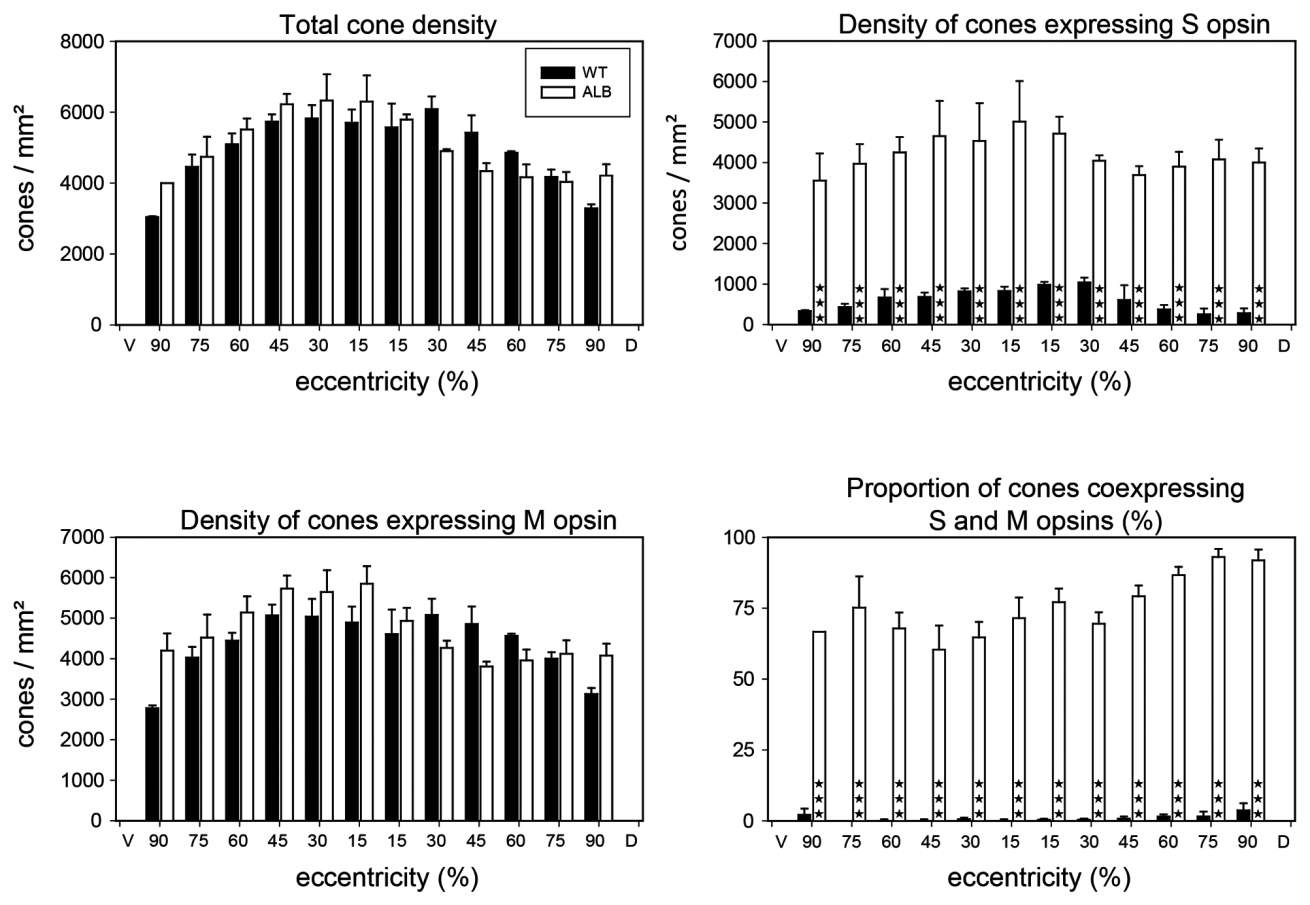
Quantitative comparison of cone densities and opsin expression in adult wildtype and albino deer mice. The local densities of cones expressing M opsin, S opsin, or both, were determined along the dorso-ventral axis of the retina in three wildtype and three albino retinae, they are given as mean and SEM. The abscissa of each graph gives eccentricity as percentage of the distance between the optic nerve head (located at 0%) and the ventral (V) or dorsal (D) margin of the retina (located at 100%), respectively. Total cone density and the density of M opsin-expressing cones are similar in both genotypes and show a density decline from central to peripheral retina. In contrast, the density of S opsin-expressing cones is much higher in the albino than in the wildtype. This is because of the large proportion of albino cones that coexpress both opsins. ★★★, differences statistically significant at p<0,001 (two-way ANOVA & Bonferroni’s post-hoc test).

The population densities of M and S cones were obtained from retinae double-immunolabeled for the S and M cone opsins (Figure 3). This provided total cone densities as well as the contributions of M and S cones. Figure 4 shows maps of the cone densities in one exemplary wildtype and albino retina, respectively. Total cone density as well as M and S cone densities displayed centro-peripheral gradients. In some retinae there was a density dip around the optic nerve head. Average total cone density in the wildtype ranged from central densities of 5000-6000/mm^2^ to peripheral densities of 3000-3500/mm^2^ (Figure 5). Hence cones constituted 1-1.5% of the photoreceptors, which is a typical proportion for nocturnal mammals. Most of the cones expressed the M opsin, with average M cone densities ranging from 4000-5000/mm^2^ in the center to less than 3000/mm^2^ in the periphery (Figure 5). The S opsin-expressing cones had central densities of up to about 1200/mm^2^ dorsal of the optic nerve head and peripheral densities of less than 400/mm^2^ (Figure 5). Depending on region, the S cones comprised 5-15% of the cones.

In adult wildtype *P. maniculatus* retinae, the M and S cones constituted separate populations with almost exclusive expression of either M opsin or S opsin in any one cone (Figure 3). On average, coexpression of M and S opsin was observed in less than 2% of the cones (Figure 5). Some retinae contained no coexpressing cones at all, in others coexpression was seen in up to 10% cones in peripheral retina. 

Average total cone densities in albino *P. maniculatus* were not significantly different from those found in the wildtype (Figure 5). The albino retina shown in Figure 4 had a particularly high cone density in central retina (7500/mm^2^). With the lower overall photoreceptor density, the cones represented a higher proportion of 1.5-2.5% of the photoreceptors in the albino. The most striking difference to the wildtype was that the majority of albino cones coexpressed the M and S opsin (Figures 3, 5). M opsin was expressed by 85-95% of the cones, which is not significantly different from the wildtype situation, but S opsin was also expressed by 75-95% of the cones (Figure 5), signifying opsin coexpression in 60-90% of the cones. The level of coexpression varied with retinal position and was particularly high in peripheral retina (Figure 5). S opsin was exclusively expressed in 5-15% of the cones (“pure S cones”), which parallels the wildtype S cone proportion. Exclusive M opsin expression was found in 5-30% of the cones.

### Postnatal development of the cone mosaic

**Figure 6:**
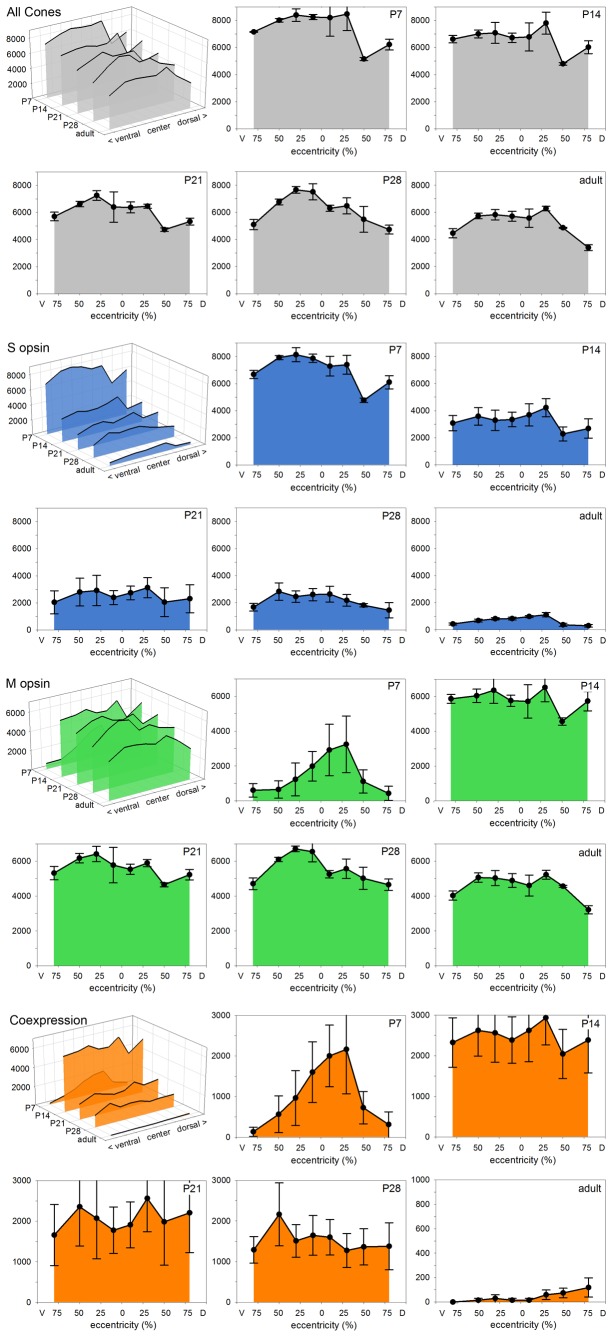
Postnatal development of cone opsin expression. The four colored blocks show the development of cone properties along the dorso-ventral axis of the retina from postnatal day P7 to adulthood. Each block contains a 3D diagram summarizing the progression of a property across the retina and over time, and the individual diagrams for each time point. For each time point, the cones were counted in three wildtype retinae double-immunolabeled for M and S opsin, data points give mean values and SEM. Top block (grey): Total cone density shows a decline with age, because of retinal areal growth. Second block (blue): Density of S opsin-expressing cones. At P7 nearly all cones express S opsin, during subsequent retinal maturation the number of S opsin-expressing cones drops dramatically to adult values. Third block (green): The density of M opsin-expressing cones is low at P7 and is highest in the central retina that leads maturation. During subsequent maturation, the number of M opsin-expressing cones increases to adult values. Bottom block (orange): Density of cones coexpressing M and S opsin, comprising practically all M opsin-expressing cones at P7 and a decreasing proportion of the cones at later stages. All vertical axes give cone densities (cones/mm²), eccentricities are given as in Figure 5.

**Figure 7:**
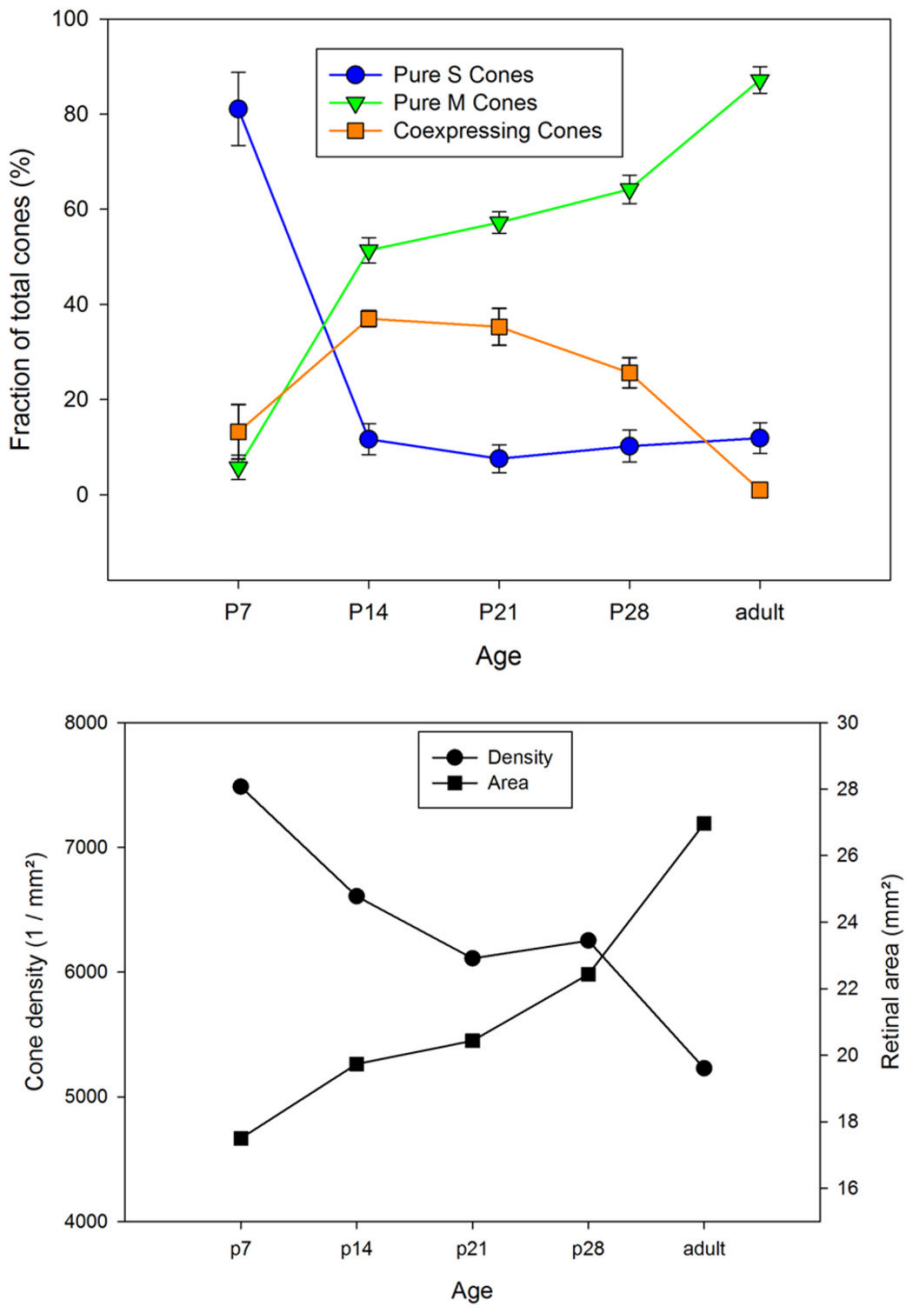
Postnatal changes of retinal size, cone density and opsin expression. Top: Cone density decreases anti-parallel to postnatal retinal areal growth. This suggests that cones are neither born nor dying after P7. Cone densities are averages across retinae without regional differentiation, retinal area at each age is the mean of three retinae. Bottom: Postnatal changes in cone opsin expression. The cones start out by expressing S opsin, and most of them subsequently switch to M opsin expression. The fraction of cones that transiently coexpress both opsins peaks around P14 to P21.

We were interested to see how the adult cone opsin expression pattern emerged during postnatal development of the retina. This could only be assessed in wildtype animals, because no young albinos were available to us. During the first few postnatal days quantification could not be done reliably because of low cone opsin expression levels and the small size of the developing cone outer segments. Hence we analyzed eyes from postnatal days P7, P14, P21, P28, and compared them to the adult pattern of 4-5 months old animals. In three retinae of each stage, the cones were labeled by opsin double-immunofluorescence, and cone types and densities were determined along a dorso-ventral transect at positions representing 10%, 30%, 50% and 80% of the distance between the optic nerve head and the retinal periphery in both dorsal and ventral direction. Relative rather than absolute distances were chosen because retinal size increases substantially during postnatal eye growth. The results are shown in Figure 6. Across the retina, highest cone densities were found at P7, ranging from >8000/mm^2^ in central to 6000-7000/mm^2^ in peripheral retina. There was a conspicuous low of about 5000/mm^2^ at 50% dorsal eccentricity that persisted up to P21. With increasing age, cone densities approached the adult values. The developmental decrease in cone density was concurrent with an increase in retinal area (Figure 7).

Figure 6 also shows the progression of opsin expression. At P7 nearly all cones expressed the S opsin, 80% of them expressed it exclusively. During the subsequent weeks, S opsin expression was continuously reduced in many cones to approach the adult S cone proportion of 5-15%. M opsin expression started around P7 in cones in central temporal retina and over the next few days spread to most cones across the retina. Hence highest densities of M opsin-expressing cones were seen at P14. This was also the time point having the highest proportion of coexpressing cones (Figure 7). After that, the density of M cones decreased with retinal expansion, whereas the proportion of pure M cones further increased as S opsin expression was terminated in many cones (Figure 7).

### S opsin tuning

 In a number of rodents the visual pigment containing the S opsin is tuned to ultraviolet (UV), whereas in others it is tuned to violet/blue. The tuning depends on a small number of strategically positioned amino acids in the S opsin (reviews [8]: [10], [11], [13], [27],). Sequencing the tuning-relevant parts of the *P. maniculatus* S opsin gene revealed the presence of those amino acids that have a crucial role in shifting the spectral sensitivity of the S pigment towards the UV range (foremost **Phe** at site 86, but also **Thr** at site 93 and **Ala** at site 97; Table 1). This suggests UV tuning of the S cone pigment in *P. maniculatus*. The sequenced parts of the S opsin were identical in wildtype and albino animals. Sequence details have been deposited in GenBank (accession number KF683088).

**Table 1:** Variation at informative amino acid sites of mammalian UV- and violet/blue-sensitive S opsins.

**Species**	**λ_max_ (nm)**	**52**	**86**	**93**	**97**	**114**	**118**
*Peromyscus maniculatus*	-	Thr	Phe	Thr	Ala	Ala	Ser
*Mus musculus*	359	Thr	Phe	Thr	Ala	Ala	Ser
*Rattus norvegicus*	358	Thr	Phe	Thr	Ala	Ala	Ser
*Sciurus carolinensis*	440	Thr	Tyr	Val	Asn	Ala	Ser
*Bos taurus*	451	Thr	Tyr	Ile	Thr	Ala	Cys

Potentially tuning-relevant amino acids of deer mouse aligned with mouse, rat, grey squirrel and ox; amino acid numbers according to bovine rod opsin nomenclature (46 bovine rho = 41 mouse S opsin). S opsin GenBank accession numbers: *Peromyscus maniculatus* KF683088, *Mus musculus* NM_007538, *Rattus norvegicus* NM_031015, *Sciurus carolinensis* DQ302163, *Bos taurus* NM_174567. Peak spectral sensitivities (λ_max_) taken from [43] for *Mus* and *Rattus* [44], for *Sciurus* [45], for *Bos*.

## Discussion

 Our quantitative photoreceptor analysis shows that the nocturnal deer mouse *Peromyscus maniculatus* has a rod-dominated retina with a low but consistent population of cones. In the wildtype deer mouse rod densities are 400,000-520,000/mm^2^, which is similar to rod densities in the nocturnal house mouse (about 440,000/mm^2^; [28]). The cones make up 1-1.5% of the photoreceptors, their density (3000-6500/mm^2^) and proportion are about half those of the house mouse (6700-15,700 cones/mm², representing ca. 3% of the photoreceptors [28]; [29],,). In the wildtype, the majority of cones express the M opsin, and a regionally varying minority of 5-15% of the cones express the S opsin. Coexpression of the opsins in individual cones is rare. This conforms to the basic mammalian blueprint (see, e.g., [4]) but is in stark contrast to the house mouse and some other mammals, where a substantial and regionally varying proportion of the cones coexpress both opsins (for rodents, see [9] [14], [15], [30],). With respect to cone proportions and cone opsin expression, the retina of the deer mouse resembles that of the rat more than that of the house mouse (rat: average 4300 cones/mm², representing ca. 1% of the photoreceptors, 5-10% of the cones express S opsin, rare opsin coexpression [31]; [32],). The deer mouse M and S cone densities reported here confirm and extend the average densities given by Shupe et al. [2].

The photoreceptor properties of the albino deer mouse differ from those of the wildtype. Whereas cone densities are similar, albino total photoreceptor densities and hence rod densities are 40% lower. This raises the cone proportion to 1.5-2.5% of the photoreceptors. Another striking difference is that a large majority of albino cones coexpress the M and S opsin (‘dual pigment cones’). As the albino retina still contains a wild-typical proportion of 5-15% ‘pure’ S cones (i. e. exclusively expressing the S opsin), it is likely that the dual pigment cones correspond to M cones of the wildtype retina. The deer mouse albino strain studied here is of the oculocutaneous albinism type 1 [33], having a mutation in the tyrosinase gene. A specific reduction of rod density by 30-40% without a reduction of cone density also has been reported for the albino ferret [34]. Similarly, rod numbers are reduced by 25-30% in adult albino BALB/c mice as compared to pigmented C57BL/6J mice [35], [36], correlating with reduced melanin levels [35]. Reduced rod density is attributed to increased rates of cell death either during postnatal rod development [35] or during early adulthood [36]. The ferret study did not assess cone opsin expression and it remains unknown whether albino ferrets show a higher incidence of dual pigment cones. A quantitative comparison of cone properties in a pigmented (Piebald Virol Glaxo) and an albino rat strain (Sprague-Dawley) showed increased S cone numbers and decreased M cone numbers in albino rats. The proportion of dual pigment cones did not differ in both strains although the adopted approach could only detect those which expressed an equal amount of both opsins and therefore likely underestimated their number, as the authors admit [32].

The albinotic phenotype somewhat resembles that of a young (1-2 weeks old) deer mouse, where transient opsin coexpression occurs. One explanation could be that the postnatal developmental signal to decrease S opsin expression in future M cones is not triggered in albinotic animals. An investigation of developing albino deer mouse retina would be required to assess the time course of opsin expression and to identify the factors influencing it. For studies of the mechanisms by which albinism affects cone opsin expression, the deer mouse appears more suitable than the house mouse, where substantial cone opsin coexpression is already present in the pigmented wildtype.

The postnatal development and maturation of deer mouse cone opsin expression is similar to that described in mouse, rat and gerbil [37]-[39]. All cones start with immunohistochemically detectable levels of S opsin expression in the first postnatal week, but most switch to M opsin expression roughly within the following week, coexpressing both opsins in the transition phase. In the deer mouse, as in the rat and gerbil, the majority of cones become pure M cones [38], whereas in ventral mouse retina the majority of initially S opsin-expressing cones maintain dominant S opsin expression and some M opsin coexpression into adulthood [15]. During retinal development, specification of cone type fate with its appropriate opsin expression is influenced by thyroid hormone and its receptor TRβ2, the retinoid X receptor γ (RXRγ), the retinoic acid receptor-related orphan receptors RORα and RORβ, and a number of other factors (recent review [40]:). However, it still remains unknown how the different, species-specific patterns of M and S cones are established. With the data base presented here, the deer mouse may be a useful auxiliary in such developmental studies.

The mammalian S cone pigment may be tuned to violet/blue or to UV. The ancestral mammalian S pigment was almost certainly UV sensitive [10], [41], and several species of rodents have retained this UV tuning (for reviews see, e. g., [4] [5], [7],). While the blue/violet sensitive S pigments present in many mammals have Tyr or Leu at amino acid position 86, the UV sensitive pigments have Phe at that critical site [8]. *Peromyscus maniculatus* has Phe at site 86, suggesting UV tuning. Although the adaptive advantage of UV sensitive vision is yet unknown, Chávez et al. [42] have suggested that it may play a role in visual communication by means of the UV reflecting urine used in scent-marking, a common behavior in most rodent species.

Concerning visual ecology, the deer mouse is a good climber and its movements in the habitat are more three-dimensional than those of the house mouse. It can be assumed that the natural horizon is a less static feature in its visual field, and that the spectral differences between skylight (containing higher proportions of short wavelengths) and light reflected from the ground are not always seen by the same part of the retina (ventral and dorsal, respectively). Hence the mouse-typical division of the retina into a ventral S opsin-dominated and a dorsal M opsin-dominated part may not have been an advantageous trait in deer mouse evolution. The exclusive expression of only M or S opsin in any one cone is expected to give the deer mouse better color vision than a mouse-like coexpression of both opsins in a large proportion of the cones.

Concerning the suitability of *Peromyscus maniculatus* as a model species for retinal studies, our results show that the cone properties of the deer mouse conform much more to the general mammalian pattern than those of the house mouse. Hence *Peromyscus maniculatus* may be better suited than mouse for the study of certain basic cone properties, including the effects of albinism on cone opsin expression. Given the same space requirements and fast generation cycles of deer mouse and house mouse, handling is easy for any facility that already works with mice. One major drawback of course is the limited availability of mutants compared to *Mus musculus*. Hence the deer mouse cannot replace the house mouse as key model species, but it can be a valuable addition.
